# Simulation of neuroplasticity in a CNN-based *in-silico* model of neurodegeneration of the visual system

**DOI:** 10.3389/fncom.2023.1274824

**Published:** 2023-12-01

**Authors:** Jasmine A. Moore, Matthias Wilms, Alejandro Gutierrez, Zahinoor Ismail, Kayson Fakhar, Fatemeh Hadaeghi, Claus C. Hilgetag, Nils D. Forkert

**Affiliations:** ^1^Department of Radiology, University of Calgary, Calgary, AB, Canada; ^2^Hotchkiss Brain Institute, University of Calgary, Calgary, AB, Canada; ^3^Biomedical Engineering Program, University of Calgary, Calgary, AB, Canada; ^4^Alberta Children’s Hospital Research Institute, University of Calgary, Calgary, AB, Canada; ^5^Department of Clinical Neurosciences, University of Calgary, Calgary, AB, Canada; ^6^Institute of Computational Neuroscience, University Medical Center Hamburg-Eppendorf (UKE), Hamburg, Germany; ^7^Department of Health Sciences, Boston University, Boston, MA, United States

**Keywords:** deep neural networks, neurodegeneration, Alzheimer’s disease, *in-silico*, cognitive computational neuroscience

## Abstract

The aim of this work was to enhance the biological feasibility of a deep convolutional neural network-based *in-silico* model of neurodegeneration of the visual system by equipping it with a mechanism to simulate neuroplasticity. Therefore, deep convolutional networks of multiple sizes were trained for object recognition tasks and progressively lesioned to simulate neurodegeneration of the visual cortex. More specifically, the injured parts of the network remained injured while we investigated how the added retraining steps were able to recover some of the model’s object recognition baseline performance. The results showed with retraining, model object recognition abilities are subject to a smoother and more gradual decline with increasing injury levels than without retraining and, therefore, more similar to the longitudinal cognition impairments of patients diagnosed with Alzheimer’s disease (AD). Moreover, with retraining, the injured model exhibits internal activation patterns similar to those of the healthy baseline model when compared to the injured model without retraining. Furthermore, we conducted this analysis on a network that had been extensively pruned, resulting in an optimized number of parameters or synapses. Our findings show that this network exhibited remarkably similar capability to recover task performance with decreasingly viable pathways through the network. In conclusion, adding a retraining step to the *in-silico* setup that simulates neuroplasticity improves the model’s biological feasibility considerably and could prove valuable to test different rehabilitation approaches *in-silico.*

## 1 Introduction

Machine learning models have emerged as essential tools for solving complex data-driven classification and regression problems in various domains, and healthcare is no exception. Many machine learning models have been developed and evaluated in the past that, for example, aim to classify if patients have neurological diseases, or aim to predict disease progression and outcomes based on clinical, imaging, and other assessment data (Lo Vercio et al., 2020; Pinto et al., 2020; James et al., 2021; Rajashekar et al., 2022). Despite their high value for computer-aided diagnosis, these machine learning models cannot be used naively as computational disease models, even when using approaches from the explainable artificial intelligence domain (Linardatos et al., 2020). However, in a more neuroscientific-inspired branch of research, deep learning models are being increasingly investigated as potential tools for modeling how the brain processes information (Kubilius et al., 2016; Yamins and DiCarlo, 2016; Lake et al., 2017; Richards et al., 2019; Lindsay, 2021). These deep neural networks are trained to mimic human behavior and function (Saxe et al., 2021). Although model architectures and training procedures are not identical to biological systems, for example by using backpropagation to learn, deep neural networks remain to be some of the best models of human-level cognition, which may provide a valuable basis for in-silico models of neurological diseases (Güçlü and van Gerven, 2014; Khaligh-Razavi and Kriegeskorte, 2014; Kaiser et al., 2017; Cichy and Kaiser, 2019; Perconti and Plebe, 2020). Establishing an *in-silico* model of neurological disease would, for example, allow us to obtain a better understanding of the effects of axonal and neuronal damage, and other pathological processes such as tau deposition on essential brain functions. Here, the term “*in-silico*” refers to the usage of computer methods for understanding biological processes in the living organism (Winder et al., 2021). Deep convolutional neural networks (CNNs), a deep learning model architecture specifically designed for solving computer vision problems such as object recognition, were originally inspired by the structure of neurons and synapses found in the mammalian visual cortex (Rawat and Wang, 2017). The concepts used to inspire CNNs date back to early models of the visual system, postulated by Hubel and Wiesel (1962, 1968). An emerging field that is gaining momentum recently involves using deep learning models as an abstraction of a healthy human brain, which can then be utilized as a basis for simulating neurodegenerative diseases (Tuladhar et al., 2021; Moore et al., 2022). Since CNNs were specifically designed for vision tasks and were modeled after information processing patterns in the mammalian brain, they can be used to model neuronal injuries that occur in the visual cortex, as for example the case in posterior cortical atrophy (PCA). PCA is characterized by the rapid deterioration and thinning of visual cortical areas such as V1, V2, V3, and V4, leading to a loss of visual recognition abilities in patients (Crutch et al., 2012; Maia da Silva et al., 2017). PCA is usually a variant of AD, caused by the same protienopathies. Previous research has established parallels between synaptic and neuronal pruning in CNNs and *in silico* models and the onset of posterior cortical atrophy (Moore et al., 2022). In this work, we compared the effects of applying either progressive neuronal or synaptic injury using an established CNN architecture (VGG19) as an initially cognitively healthy model. The CNN was trained to perform object recognition on 2D images, akin to the Boston Naming Test (BNT) or other similar neuropsychological assessments testing visual function (Williams et al., 1989). During the BNT, patients are presented with stimuli in the form of line drawings of items of 60 categories and are asked to identify the objects. Therefore, it may be possible to draw parallels between object recognition tasks of the CNN and cognitive assessments such as the BNT.

**Figure 1:**
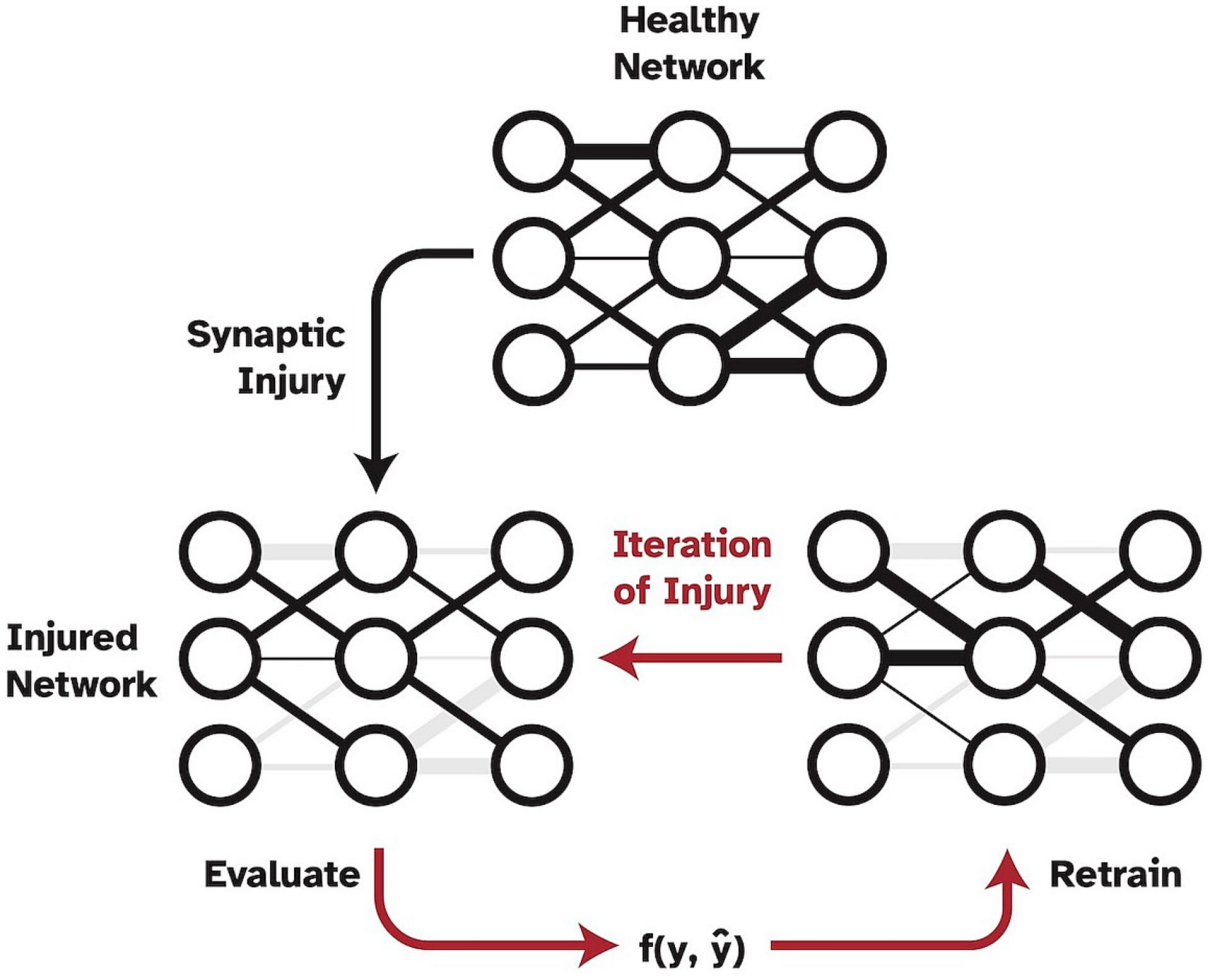
Pipeline of progressive synaptic injury with the added mechanism of neuroplasticity. After each iteration of synaptic damage, the model is retrained on the original training split of data and evaluated. Ablated synaptic weights are shown in grey.

However, a shortcoming of this work was the method in which injury was applied to the network, which was not biologically realistic. Specifically, injury was progressively and statically imposed, without allowing the model to update weights or be exposed to any new training data. Thus, the aim of the present study was to expand upon and improve Moore et al.’s (2022) work by adding the crucial mechanism of simulated neuroplasticity via retraining as shown in Figure 1. In the present study, synapses are specifically set to zero to simulate full synaptic death in the visual cortex. While other pathological mechanisms may precede synaptic death and lead to a functional decline in synapses over time, synaptic death is the ultimate effect of any dementia disease. The ability of the human brain to develop new synapses is very limited in adults so that the remaining synapses need to be retrained to account for the loss and as a means of neuroplasticity. Thus, in this study, we froze the injured weights to prevent them from being subjected to the retraining process to simulate disease effects in humans where dead synapses cannot be simply replaced by new ones (John and Reddy, 2021). Furthermore, one could argue that a standard VGG19 network is overparameterized, and thus has too much reserve capacity as compared to human cognitive reserve, to be a biologically realistic in-silico model when studying injuries. Therefore, in the present study, we investigate two different models as a baseline for cognitively healthy object recognition. The analysis was performed using a full VGG19 model as well as using a highly pruned version of the VGG19 to examine the effects of plasticity as a function of imposed injury and number of model parameters or synapses.

While model compression and pruning are active branches of deep learning research, our paradigm of ‘injury’ does not follow typical pruning methods, which aim to reduce the number of parameters in a model while retaining full function (Choudhary et al., 2020). In contrast, we use progressive random pruning followed by retraining to simulate the cognitive effects of a neurodegenerative disease as a function of abnormal levels of atrophy. We find that by adding iterative retraining with every pruning step of synaptic ablation, the decline of visual cognition is much smoother and more similar to what is seen in patients with Alzheimer’s disease (AD) (Mattsson et al., 2017).

## 2 Materials and methods

### 2.1 Models and data

The basis for the cognitively healthy object recognition model is a VGG19-like model with batch normalization trained on the CIFAR10 dataset (Russakovsky et al., 2015). CIFAR10 is a commonly used dataset for computer vision research, which consists of 60,000 32 × 32 natural color images. The dataset consists of 10 classes: plane, car, bird, cat, deer, dog, frog, horse, ship, and truck, with 6,000 images in each class. The train/test split used is 50,000 and 10,000 images, respectively. This dataset was chosen due to the relative simplicity and ease of computational load. The model architecture used in this work is comprised of five convolutional blocks, each followed by a batch normalization layer, ending with four max-pooling layers, and finally, a Softmax activation with 10 nodes corresponding to the 10 classes in the dataset. Our model was pretrained on ImageNet and fine-tuned on CIFAR10 for 100 epochs with a learning rate of 0.001, using a batch size of 128, and a stochastic gradient descent optimizer with momentum 0.9. After training, the full model achieves an accuracy of 93.74% on the test set of images. A VGG19 model was chosen for this research as it has been to have high correlation with mammalian neuronal activation data and is widely accepted as a SOTA baseline model in computer vision tasks.

Previous research has shown that VGG19 models may be largely overparameterized, especially for classifying CIFAR10, due to their retention of high levels of accuracy when subjected to optimized pruning techniques (Frankle and Carbin, 2018; Ayinde et al., 2019). More specifically, they likely have learned unnecessary or redundant pathways due to the enormous number of synapses and neurons they are equipped with. The brain has also been shown to be overparameterized, but is likely much more constrained by energy usage and physical space (Drachman, 2005; Mizusaki and O’Donnell, 2021). Thus, to investigate potential spurious results that are driven by overparameterization, rather than model plasticity abilities, and to perform experiments in a more physically constrained setting, we also investigated a considerably more optimized compressed model. To this end, we performed structured model pruning on the trained full VGG19 model. Model compression was informed by graph dependencies using methods developed and described in Li et al. (2016) and Fang et al. (2023). Filters and associated weights were removed simultaneously based on their L1 norm until the model inference speed, in terms of floating-point operations (FLOP), was increased by a user-defined amount. To probe the amount of structured pruning the model could tolerate before significant declines in accuracy, we performed model compression multiple times. The compression resulted in models that had been sped up 2x, 3x, and 4x from the original inference speed while maintaining similar, high accuracies. We found that increasing FLOP by three times with respect to the original VGG19 model resulted in a compressed model with only 8.54% of the original weights. Despite this considerable reduction of weights, this compressed model retained an accuracy of 93.3% on the test set. All model injury and retraining experiments described below were performed on both the full VGG19 and this compressed version. Experiments were conducted using Pytorch 1.13 on an NVIDIA GeForce RTX 3090 GPU with 24GB of memory.

### 2.2 Synaptic ablation and retraining

Synaptic ablation was imposed on the network in a uniformly disperse and progressive manner as originally proposed in Moore et al. (2022). This ‘injury’ type was implemented by setting weights from convolutional layers and dense layers in the network to zero, effectively severing the connections between nodes. This approach is akin to progression of synaptic damage seen in neurological diseases that accelerate atrophy rates in the brain, such as posterior cortical atrophy. It should be noted that this synaptic injury and retraining is not the same as optimized model pruning, and thus is more biologically reasonable as an in-silico paradigm. We imposed random synaptic ablation at a step rate of $1-{\left(1-\gamma \right)}^{n}$ where γ is the relative fraction of weights being ablated to the remaining uninjured weights in the network, and n is the number of iterations of injury. Once a synapse is ablated, it can no longer be used by the model and is excluded from the retraining process.

In our experiments, γ was set to 0.2 (20% of weights ablated) and n was set to 15 iterations as this was found to be representative of injury resolution while maintaining reasonable computational requirements. Following each iteration of injury, we retrained the model on the training split of data using the same initial training parameters for three epochs to investigate how model performance could be regained. With each retraining step, the optimizer was reinitialized while the injured weights remained set to zero so that the model had to find alternative pathways to regain test performance. We performed this analysis ten times to reduce the risk that biasing effects related to the order in which synapses were randomly ablated are affecting the results.

### 2.3 Representational dissimilarity matrices

Representational dissimilarity matrices (RDMs) were computed to examine the changes in internal activations and representations of categorized data of both the injured and retrained networks when compared to the respective baseline, healthy networks. RDMs are routinely used to quantitatively correlate brain-activity, behavioral measurement, and computational modeling (Kriegeskorte et al., 2008; Khaligh-Razavi and Kriegeskorte, 2014; Mehrer et al., 2020). RDMs measure the representational distance between two sets of model activations given different inputs and can be used to visualize representational space. RDMs were generated by pairwise comparison between activations of the network’s penultimate layer for all test set images using Pearson’s correlation coefficient. We constructed RDMs for each iteration of both network ablation and retraining, and then compared them to the healthy network’s RDM using Kendall’s tau correlation coefficient. This approach effectively enabled us to quantify the effects of both injury and retraining on internal activations of the networks. Comparison of RDMs of the model as it is progressively injured and retrained allows for the examination of how the relative structure of representational space is affected and reconstructed with injury and retraining.

### 2.4 Brain-score

The Brain-Score is a widely used metric that has been developed to analyze how the CNN model activations are correlated and predictive of mammalian neural activation data (Schrimpf et al., 2018, 2020). Within this context, VGG19 has been found to be a relatively highly ranked model in terms of neural predictivity. We wanted to investigate how imposing injury to a ‘healthy’ VGG19 model affected its Brain-Score. Therefore, we created our baseline Brain-Scores by following the methods outlined by Schrimpf et al. (2018) and used the publicly available neural recording benchmarks for visual areas V1, V2, V4, and IT (Freeman et al., 2013; Majaj et al., 2015). The neural recordings dataset contains macaque monkey neural responses to 2,560 naturalistic images. More detail on this data and the methods we used to calculate Brain-Score can be found in the publicly available code from Schrimpf et al. We used the neural benchmarks to establish how well the internal representations of our CNN models matched internal representations of mammals. In computing the Brain-Score, we compute a composite measure of neural predictivity scores for all aforementioned visual areas. Neural predictivity is evaluated on how well the responses, or internal activity in our CNNs predicted the neural activity in the biological neural recordings. Consistent with the literature, we performed this analysis using principal components analysis to reduce the dimensionality of model activations to 1,000 components, and then used partial least squares regression with 25 components to correlate the CNN model activation to mammalian neural activations. Correlation coefficients were calculated for each of the publicly available benchmarks and then averaged to calculate an average Brain-Score. Brain-Score values were analyzed for progressive iterations of injury and retraining in the models. It should be noted that only publicly available, neural benchmark datasets were used in our Brain-Score calculations so there were disparities between our ‘healthy’ VGG19 Brain-Score and that reported on the Brain-Score leaderboard.

## 3 Results

### 3.1 Accuracy

**Figure 2:**
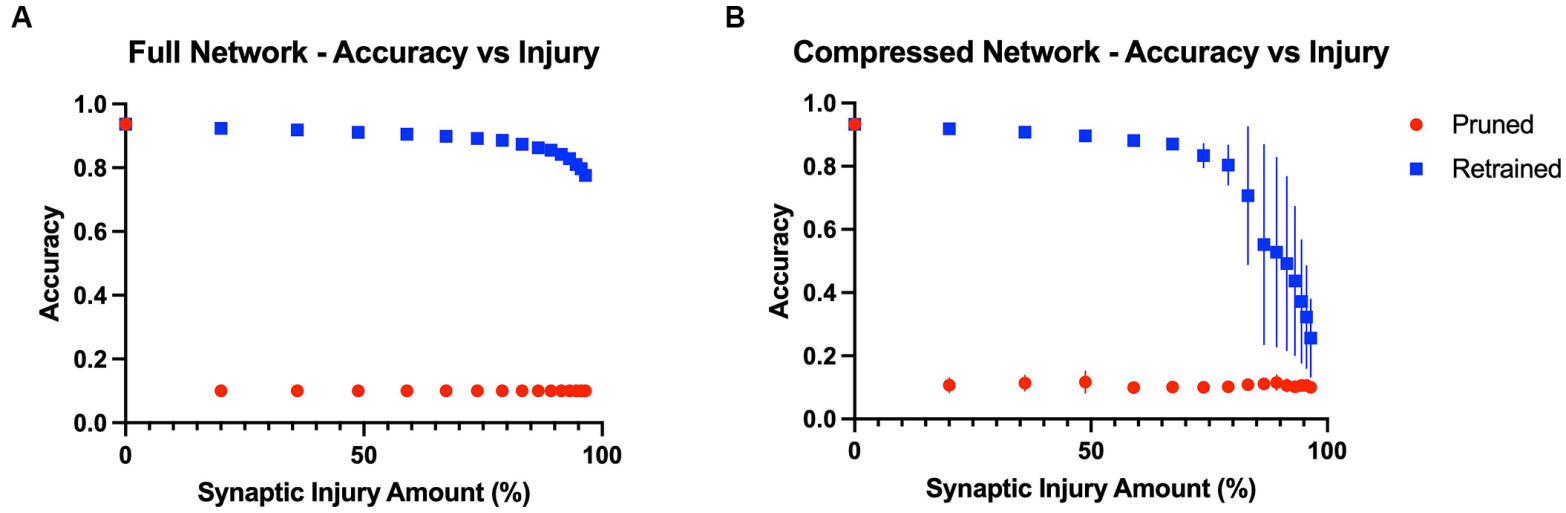
Model accuracies as a function of progressive injury and retraining. **(A)** Model accuracy and standard deviations as a function of progressive synaptic damage and retraining for the full VGG19 model. The standard deviations in accuracy of the full model are extremely low (*~* ± *0.005%*). The model immediately has a substantial drop in accuracy after 20% of the synapses are removed but regains close to complete function after retraining on the training set. Substantial levels of accuracy are regained with the addition of retraining, even at extremely high levels of injury. **(B)** Compressed model accuracy as a function of synaptic damage. Retraining leads to large gains in accuracy until injury levels of 85% and higher, at which point the model shows a steeper decline in accuracy even with retraining.

Baseline model accuracies were 93.7 and 93.3% for the full VGG19 and compressed VGG19, respectively. The results showed that model performance was immediately affected by the first application of random synaptic injury (20% of synapses randomly deleted), leading to a large drop in object recognition accuracy in both the full and compressed models across all classes of the test split of the CIFAR10 benchmark dataset. Quantitatively, after the first iteration of injury, the full model’s accuracy on the test set suffered a drop from 93.7% to a mere 10.0%, essentially chance level. Interestingly, the accuracy was substantially restored to 92.4% ± 0.001% after three epochs of the retraining iteration. With each iteration of injury, this pattern of large accuracy drops continued to repeat, with the model again tending to perform only at chance level (10% accuracy). However, retraining continued to improve model accuracy by a large margin, even until 96.5% of initial synapses had been removed. After this point, the model could only regain accuracy levels of 77.6% ± 0.010% with retraining. These effects are shown in Figure 2A. Similar to the full model, the compressed model also proved to be largely affected by introducing plasticity to the injury paradigm. With each iteration of synaptic injury, the compressed model accuracy plummeted to chance level accuracy. Remarkably, even with the initial healthy network containing a mere 8.54% of the size of the full VGG19, on average the compressed model was able to regain high levels of object recognition accuracy after retraining. Even after 48.8% of synapses were injured, the compressed model recovered 89.6% ± 0.005% accuracy with the retraining iteration. At injury levels of 83% and higher, the compressed model exhibited large standard deviations in accuracy over the ten trials that were performed (~ ± 20%) (Figure 2B). This may be due to the extreme synaptic sparsity that is associated with high injury levels. If a highly salient pathway in the model with limited synapses is ablated, the model may not be able to recover accuracy with retraining.

To further investigate the relationship between model size and recovery with retraining, we examined accuracy levels based on the total number of parameters within the full and compressed models. We inspected the point in the injury progression where, after retraining, the two models displayed close to identical levels of accuracy, and after which the retrained accuracies no longer displayed similar levels of accuracy. This point was found after the full model (20.04 million parameters) had been injured so that 83.2% of synapses had been removed, leaving the model with 3.36 million parameters. After retraining, the full model showed accuracy levels of 87.4% ± 0.003% on the test set. The point at which the compressed model showed similar levels of accuracy (87.0% ± 0.011%) was after 67.2% of its original synapses had been injured. This level of injury left the compressed model with a mere 0.560 million parameters. Furthermore, we assessed accuracy levels when the two models contained a similar number of parameters. This occurred when the full model had been injured by 96.5%, and thus had 0.705 million parameters, and when the compressed model had been injured by 59.0% and had 0.700 million parameters. The accuracy levels were significantly different, at 77.6% ± 0.010 and 88.1% ± 0.010%, respectively. These results indicate that model size and overparameterization are not the sole contributing factors to the impact adding plasticity has on the degenerative *in-silico* paradigm.

### 3.2 Representational dissimilarity matrices

**Figure 3:**
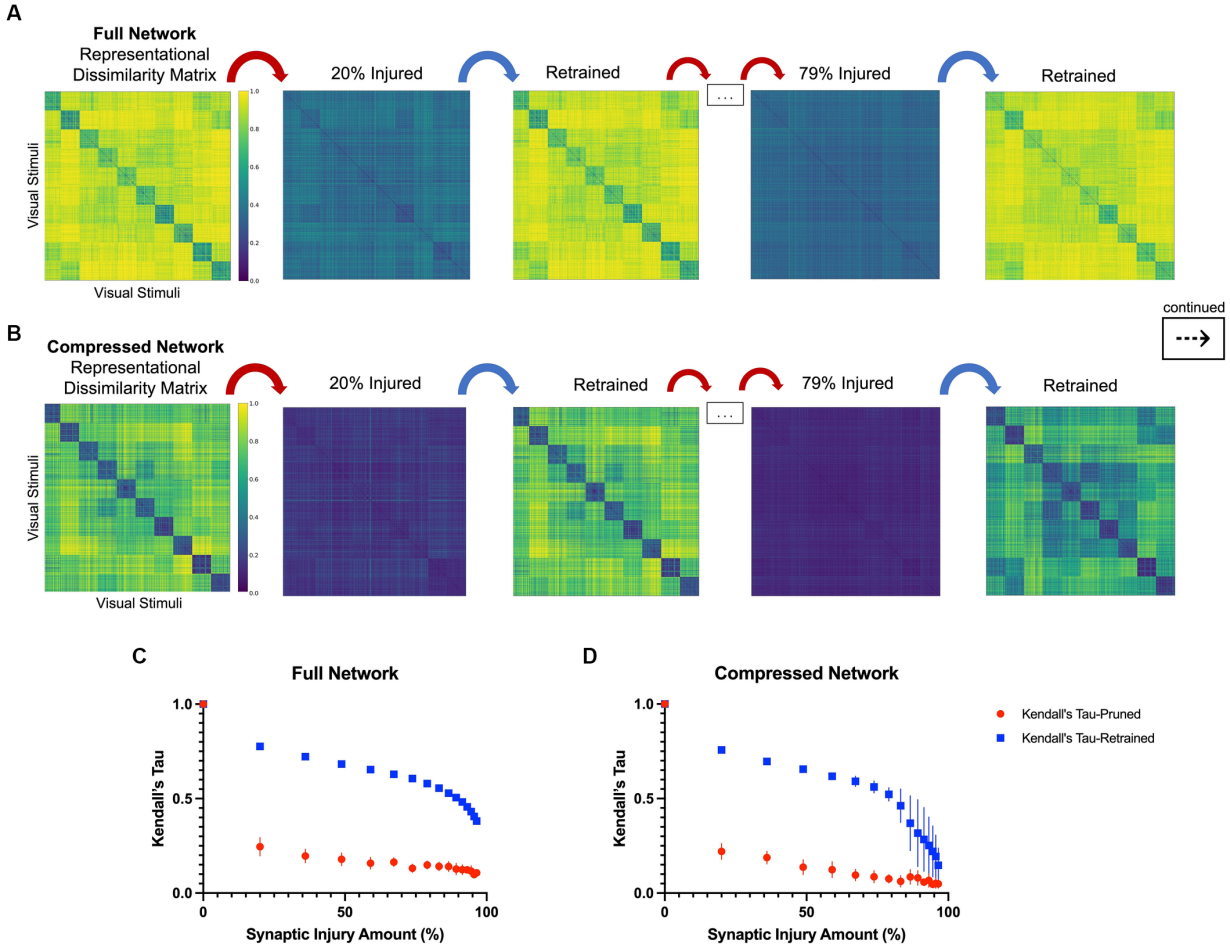
Representational dissimilarity matrices with injury and retraining, as described in Section 2.3. **(A)** A qualitative examination of representational dissimilarity matrices as an initial iteration and a later iteration of injury and retraining are completed in the full model and **(B)** in the compressed model. Before injury, both models have distinct activations for each of the 10 different classes in CIFAR10. **(C)** Kendall’s tau correlation as a function of synaptic injury in the full model. As injury and retraining are imposed, the activations first lose their categorical nature, but are able to recover some of it with retraining. **(D)** Kendall’s tau correlation as a function of synaptic injury for the compressed model. A similar trend is seen to that in the full model albeit there is larger variance in correlation values with higher levels of injury.

In line with the results of the model accuracy evaluation, the internal representations of the models were able to regenerate and recover with retraining after injury. In the first iteration of injury and retraining (20% of synapses ablated) on the injured full model, the correlation to the healthy RDM revealed a Kendall’s tau of 0.25 ± 0.05. After retraining for three epochs, the model was able to reconstruct activations more similar to those of the healthy model, resulting in a Kendall’s tau value of 0.78 ± 0.01. Comparatively, the compressed model’s internal activations also degraded after the first iteration of damage and showed a Kendall’ tau value of 0.22 ± 0.04. Upon retraining, the internal activations displayed an increased correlation to the healthy activations that resulted in a Kendall’s tau of 0.76 ± 0.02. Figures 3C,D show how retraining after each injury step led to regaining category-distinguishable activations and a smooth cognitive decline. A qualitative examination also reveals how the network activations were affected through injury and retraining. As seen in Figures 3A,B, the uninjured networks initially had clearly defined activations grouped according to object classes in the CIFAR10 dataset. Upon injury, the networks lost this categorical representation and the RDMs became noisy. After retraining, however, categorical structure between the classes was regained. This trend continued progressively as injury and retraining steps were applied to the full network, but at high levels of injury, there came a point where there was no longer a difference in Kendall’s tau correlation between injured and retrained RDMs (e.g., at injury levels higher than 95%).

### 3.3 Brain-score assessment

The Brain-Score was computed for progressive steps of injury and retraining for both models (Table 1). Brain-Scores are reported as the mean Brain-Score of the four brain regions (V1, V2, V4, and inferior temporal (IT) cortices) that were used in the correlation analysis. The averages are reported with the standard deviations. Overall, it was found that Brain-Scores decreased in value as synaptic injury increased. Thus, the injured models tended to be less ‘brain-like’ than both of the healthy models (i.e., the uninjured full and compressed models) according to the scores. Following retraining, the models regained a level of Brain-Score comparable to healthy models. These finding indicate that adding retraining allows models to retain ‘brain-like’ features in terms of internal activations, while still exhibiting functional deficits (i.e., loss of object recognition abilities).

**Table 1:** Brain-Scores are reported for the injury and retraining steps as injury level increases.

Full network									
Injury amount	0% (healthy)	20%	48.8%	67.2%	79.0%	86.6%	91.4%	94.5%	96.5%
Brain score (injured)	0.342 ( ± 0.028)	0.335 ( ± 0.021)	0.329 ( ± 0.024)	0.318 ( ± 0.025)	0.309 ( ± 0.021)	0.312 ( ± 0.021)	0.320 ( ± 0.020)	0.244 ( ± 0.017)	0.278 ( ± 0.021)
Brain score (retrained)		0.341 ( ± 0.027)	0.345 ( ± 0.028)	0.348 ( ± 0.027)	0.341 (0.024)	0.336 ( ± 0.023)	0.349 ( ± 0.024)	0.338 ( ± 0.024)	0.342 ( ± 0.025)
Compressed network									
Brain score (injured)	0.343 ( ± 0.028)	0.291 ( ± 0.025)	0.290 ( ± 0.024)	0.276 ( ± 0.018)	0.279 ( ± 0.018)	0.314 ( ± 0.020)	0.258 ( ± 0.020)	0.267 ( ± 0.020)	0.266 ( ± 0.017)
Brain score (retrained)		0.348 ( ± 0.026)	0.342 ( ± 0.023)	0.343 ( ± 0.022)	0.346 (0.026)	0.342 ( ± 0.025)	0.335 ( ± 0.025)	0.320 ( ± 0.024)	0.300 ( ± 0.020)

The Brain-Score values tend to decrease as injury is progressively applied, but become comparable to the healthy models’ Brain-Scores with retraining.

## 4 Discussion

### 4.1 Main findings

The proposed framework for *in-silico* modeling of visual impairments associated with neurological diseases and retraining to model neuroplasticity may lead to improved disease understanding. With further development, we may establish more biologically realistic computer models that can be injured in different ways, instead of having to collect data from hundreds of patients with different disease patterns to obtain similar information. Furthermore, the development of this branch of research may also enable us to investigate the benefit of potential interventions to re-learn specific brain functions, for example, cognitive rehabilitation therapies. This work specifically enhances the feasibility of these models by including neuroplasticity in the simulated disease progression. It was found that this approach leads to a more biologically relevant pattern of cognitive decline with respect to the load of injury. The human brain has remarkable abilities to reorganize pathways, develop new connections, and arguably even create new neurons, typically referred to as neuroplasticity or as the neurocognitive reserve (Esiri and Chance, 2012). Simply damaging a network all at once without allowing it to retrain in between or during injury ignores this important ability of the human brain. Previous works using CNNs to model neurodegenerative diseases used a static injury paradigm that led to extreme loss of object recognition abilities even with low levels (i.e., 15–20%) of synapses injured (Lusch et al., 2018; Tuladhar et al., 2021; Moore et al., 2022).

The main finding of the current study is that with the incorporation of retraining to simulate neuroplasticity after the progression of injury, the models’ object recognition abilities progressively decline at a much smoother and slower rate than without retraining. This slow decline is more akin to the degradation of cognitive abilities seen in patients with AD and it’s PCA variant (Hodges et al., 1995; Fox et al., 1999; Jefferson et al., 2006) than the decline patterns previously observed. Expanding upon this previous research simulating statically imposed injury, here we developed a framework that is able to simulate irreversible injury, while the unaffected filters and weights were subjected to ‘re-learning’ processes to stimulate reorganization of the information flow that makes use of existing reserve capacities in the injured model. We found that the retrained models were able to compensate for the damaged pathways (synapses) and reconstruct the original activation patterns of the healthy models to a large extent when presented with images in the test set. Additionally, in this work we validate that this ability was not a direct function of initial model size. Generally, it is reasonable to expect that after being injured, an overparameterized model may exhibit large gains in task performance with retraining. However, here we show that a model that is much more compressed, and thus highly optimized in terms of number of parameters, displays remarkably similar abilities to re-gain task performance using increasingly minimal available pathways through the network, which is more similar to the human brain. Thus, we believe that the introduction of the biologically important concept of neuroplasticity, which equips our CNNs with a retraining mechanism, can be seen as an important step toward developing biologically more meaningful *in-silico* models of neurodegenerative diseases and other injuries of the human brain.

### 4.2 Limitations and future work

One important limitation of this work is related to the notable differences in information processing between CNNs and the biological visual system (Lonnqvist et al., 2021) (e.g., convolutional filters are global in a CNN while the human visual system also has filtering units that are responsible for certain parts of the receptive field). However, while this remains to be true, the object recognition performance of CNNs is comparable to that of humans, and CNNs have the ability to predict neural activation in the primate visual cortex better than any other computational model to date (Cadieu et al., 2014; Khaligh-Razavi and Kriegeskorte, 2014; Yamins et al., 2014). While a CNN works very differently at the neural level, the general organization is broadly representative of a visual network with a hierarchy of connections. We see this work as utilizing the similarities between CNNs and the visual cortex to further develop the feasibility of using deep learning models as an *in-silico* model for neurodegenerative diseases. The success of convolutional neural networks for predicting neural activity in the visual cortex makes them excellent models for modelling visual cognition. In theory, the setup presented in this work can be extended to other brain regions and cognitive or motor functions. For example, language models could be used to investigate how lesions in the auditory and frontal cortex affect language function. However, it should be noted that more research is probably needed first to investigate how similar other deep learning models for other tasks are to the human brain akin to the comparably extensive research investigating the biological feasibility of CNNs. Furthermore, while CNNs are well accepted models of the human visual system, there may be opportunities to increase the similarities to the human brain even more (Lake et al., 2015). Future work may be extended to simulate different neural damage, such as more localized lesions to model conditions like cerebral stroke or multiple sclerosis.

As previously mentioned, patients with AD often undergo cognitive assessments that probe visual object recognition abilities and recall (i.e., the Boston Naming Test). Such visual assessments together with longitudinal, high-resolution MRI data to assess atrophy could be used in future to optimize and validate the proposed in-silico model of AD but is outside the scope of this work.

Crucial future directions for this work will be to further investigate the details surrounding the iterative retraining process, as well as more realistically represent disease progression. Such investigation will allow for the exploration of rehabilitation strategies in terms of what methods of retraining enable in-silico models to regain the most function. Additionally, we can provide models with training data that are directly related to the types of errors the models begin to make with initial injury. This could be compared against re-training strategies that would simply re-use all initial training data. In addition, it may be important to evaluate the effects of other variables such as training the network on new data rather than previously seen data, or adjusting the number of epochs used in one iteration of retraining. Some studies have identified that specific task-oriented cognitive training strategies (i.e., face recognition practice) show higher memory related brain activity and task performance for patients with Alzheimer’s disease (Cotelli et al., 2006; Choi and Twamley, 2013). Notably, it may be possible to model different pathological processes of AD by gradually decaying weight values to zero rather than fully removing synapses in a single iteration. This could, for example, be used to simulate the accumulation of hyperphosphorylated tau, which is often assumed to precede synaptic death.

By probing these types of differences in network plasticity and recovery, it may be possible to identify optimal intervention strategies and relate these findings to rehabilitation techniques used in patients with dementia. This study lays further groundwork toward using deep learning models to effectively simulate disease progression, with (bright) potential to develop cutting edge in-silico models.

## Data availability statement

The original contributions presented in the study are included in the article/supplementary material, further inquiries can be directed to the corresponding author.

## Author contributions

JM: Conceptualization, Formal analysis, Investigation, Methodology, Software, Visualization, Writing – original draft. MW: Conceptualization, Methodology, Writing – review & editing. AG: Software, Writing – review & editing. ZI: Conceptualization, Resources, Writing – review & editing. KF: Methodology, Visualization, Writing – review & editing. FH: Conceptualization, Visualization, Writing – review & editing. CH: Conceptualization, Writing – review & editing. NF: Conceptualization, Supervision, Writing – review & editing.
